# The comparison of the repair bond strength of the composite resin to direct and indirect composite restorations with different surface preparations

**DOI:** 10.34172/joddd.2023.35422

**Published:** 2023-07-17

**Authors:** Hasibe Sevilay Bahadir, Selin Polatoğlu, Duygu Tuncer, Çiğdem Çelik

**Affiliations:** ^1^Department of Restorative Dentistry, Ankara Yıldırım Beyazıt University Faculty of Dentistry, Ankara, Turkey; ^2^Specialist in Restorative Dentistry, Private Clinic, Ankara, Turkey; ^3^Department of Restorative Dentistry, Kırıkkale University Faculty of Dentistry, Kırıkkale, Turkey

**Keywords:** Adhesive, Bond strength, Composite resin, Ceramage, Indirect composite, Silane

## Abstract

**Background.:**

Indirect restorations have been employed in restorative dentistry to solve some of the drawbacks of direct restorations. The aim of this study was to evaluate the effect of different modes of a universal adhesive resin on the repair capacity of two indirect resin composites and a direct resin composite.

**Methods.:**

Indirect composite resins (Ceramage and Gradia Plus) and a direct composite resin (Filtek Z250) were prepared in a plastic mold with a height and diameter of 2-mm and 6-mm, respectively. Composite blocks were thermocycled (5000 cycles, 5°C-55°C). Then, according to their surface treatments, composite blocks were categorized into six-groups: Group 1: ER (etch&rinse), Group 2: SE (self-etch), Group 3: Bur+ER (bur+etch&rinse), Group 4: Bur+SE (bur+self-etch), Group 5: Bur+Silane+ER (bur+silane+etch&rinse), Group 6: Bur+Silane+SE (bur+silane+self-etch), respectively. After surface treatments and adhesive application for bonding with a direct resin composite, all groups were then thermocycled before performing shear-bond-strength-test. Failure modes were evaluated using a stereomicroscope. Data were analyzed by two-way-ANOVA and Bonferroni-test (*P*<0.05).

**Results.:**

The highest bond-strength values were obtained for Bur+Silane+SE groups, while the lowest values were obtained for the Bur+Silane+ER groups for all materials. Statistically significant differences were observed between the Bur+Silane+ER group and ER, Bur+ER and Bur+Silane+SE groups in Gradia Plus (*P*<0.05).

**Conclusion.:**

The self-etch-mode of the universal-adhesive and silane applications led to the increase in the repair-strength of the adhesive in the Filtek Z250 and Ceramage. The self-etch-mode of the universal-adhesive might be used to reduce adhesive-application-steps in the clinical repair procedures.

## Introduction

 There is an increase in the number of tooth-colored restorations due to esthetic demands of patients as well as developments in adhesive dentistry. As extensively used restorative materials, resin composites have become the first choice for direct restorations by clinicians.^1^ Indirect restorations have been employed in restorative dentistry to solve some of the drawbacks of direct restorations, such as polymerization shrinkage and difficulty in establishing optimum proximal contact creation and anatomic form.^2-4^ Indirect resin composites are referred to as laboratory composites, which are used to fabricate several restorations such as inlays, onlays, and overlays, etc. First and second generations of indirect composites were developed in the early 1980s and mid-1990s, respectively. The clinical performance of second-generation indirect composite resins is better^5^ than those of advanced mechanical and older-generation composites,^3^ with their high filler content of ~66%. For the polymerization of indirect composites in the laboratory, special devices have been produced using light, heat, pressure, and their combination to increase the durability and longevity of composites.^5^

 Despite the advantages of indirect resin composites, clinical problems, including bulk failure, chipping, discoloration, and wear, may occur over time, and the restoration needs to be replaced or repaired to restore its function and aesthetics.^4,5^ In such cases, the intraoral repair of restorations is typically preferred compared to replacement. This procedure is not only less invasive but also cost-effective, with low pulp irritation.^4,6^ The appropriate bonding between the aged indirect composite and the novel direct composite is achieved during the repair process by mechanical roughening the old one’s surface and the application of chemical agents as a conditioner.^4^ Mechanical roughening is achieved by the use of diamond bur, etching with phosphoric acid or hydrofluoric acid, air abrasion, laser irradiation, sandblasting, and silane application. In addition, etch & rinse, self-etch, and universal or multimode adhesive systems are utilized alone or in combination.^7^ Universal adhesives exhibit considerably wide applications, and depending on the specific clinical situation and personal preferences of the operator, a single-bottle, no-mix adhesive system can be ideally used in the etch & rinse, self-etch, or selective-etch mode.^8^ Universal adhesives can also be used to place direct and indirect restorations and are compatible with self-cured, light-cured, and dual-cured resin-based cements, according to manufacturers.^5^

 Numerous in vitro studies utilizing different surface treatments and different adhesive systems and silanes have been reported previously.^3,7,9-12^ Unfortunately, universal evidence regarding the appropriate techniques and materials to be used is not available.

 In this study, the effect of different modes of a universal adhesive resin (viz. I-Bond Universal) on the repair capacity of a direct resin composite and two indirect resin composites were investigated. In addition, surface treatment methods as well as effects of silane on the bond strength of materials were examined. The tested hypotheses were as follows:

- H1: The repair capacity of the tested materials was not affected by different surface treatment procedures.
- H2: A significant difference in the repair capacities of direct and indirect composite resin materials was not observed.

## Materials and Methods

 Two indirect composite materials, viz. Gradia® Plus and Ceramage, respectively, and a direct composite material, viz. Filtek^TM^ Z250, were used herein. Table 1 lists the manufacturers and material compositions.

**Table 1:** Components of materials used in the study

**Materials**	**Type**	**LOT number**	**Manufacturer**	**Main component**
Gradia ® Plus	Indirect composite	1901151	GC Inc., Kyoto, Japan	UDMA, EDMA (75 wt% filler: Ceramic, Prepolymer, SiO2)
Ceramage	Indirect composite	121828	SHOFU Inc., Kyoto, Japan	UDMA, UDA, zirconium silicate (73%wt), Pigments and others
Filtek^TM^ Z250	Direct composite	N968746	3M ESPE, St. Paul, MN, USA	Organic matrix: TEGDMA < 1–5%; Bis-GMA < 1–5%; Bis-EMA 5–10%; UDMA 5–10% Fillers: Zirconia/silica; 60 vol% inorganic fillers
i-BOND universal	Universal adhesive system	K010034	HERAEUS-KULZER, Hanau, Germany	UDMA, 4-META, MDP glutaraldehyde, acetone, water, photo-initiators, stabilizers
Dentobond	Silane	4178-39PFXS	ITENA, Paris, France	Etilakol 97%, glisidokspropitrimetoksisilan 3%
Condac 37 Acid-etch	37% Orthophosphoric acid	180118	FGM, Joinville, Brazil	37% phosphoric acid, thickening agent, coloring agent, deionized water
Red code diamond fissure bur	Diamond bur	01304	G&Z Instrumente GmbH, Lustenau, Austria	Fine grain size

Abbreviations: Bis-GMA, Bisphenol A diglycidyl ether dimethacrylate; Bis-EMA, Bisphenol A Polyethylene Glycol Diether Dimethacrylate; TEGDMA, Triethylene Glycol Dimethacrylate; UDMA, Diurethane dimethacrylate; 4-META, 4-methacryloxyethyltrimellitic acid anhydride; EDMA, ethyleneglycoldimethacrylate; EMA, ethylenemethacrylate, MDP, 10-methacryloyloxydecyl dihydrogen phosphate

###  Experimental design

 In this study, a total of 270 specimens were prepared (n = 90) by placing a 2-mm layer composite in transparent plastic molds with a height of 2 mm and a diameter of 6 mm. After the placement of the resin composite, the top surface was covered with a Mylar strip and compressed with a glass plate to obtain a smooth surface. Then, the direct composite resin specimens were light-cured for 20 seconds using an LED curing unit. To finish polymerizing indirect composites, specimens were placed in a laboratory light curing unit for 3 minutes, as directed by the manufacturer. All specimens were removed from the plastic mold after polymerization and kept in distilled water at 37°C for 24 hours. To achieve standard surfaces, the specimens were polished using silicon carbide papers of 600, 800, and 1200 grit. Then, all direct and indirect composite specimens were embedded in acrylic blocks and thermocycled 5000 times in water baths between + 5°C and + 55°C with a rest time of 20 seconds in each bath (Esetron Smart Robotechnologies, Ankara, Turkey). Following this procedure, all specimens were randomly divided into the following six subgroups for different surface treatments (n = 15):

- Group 1: ER [universal adhesive’s etch and rinse mode (i-BOND universal, Heraeus-Kulzer, Hanau, Germany)]
- Group 2: SE [universal adhesive’s self-etch mode]
- Group 3: Bur + ER [roughen with bur (G&Z Instrumente GmbH, Lustenau, Austria) + universal adhesive’s etch and rinse mode]
- Group 4: Bur + SE [roughen with bur + universal adhesive’s self-etch mode]
- Group 5: Bur + Silane + ER [roughen with bur + acid etching (Condac 37 Acid-etch, FGM, Joinville, Brazil) + silane (Dentobond, Itena, Paris, France) + universal adhesive]
- Group 6: Bur + Silane + SE [roughen with bur + silane + universal adhesive’s self-etch mode]

**Figure 1:**
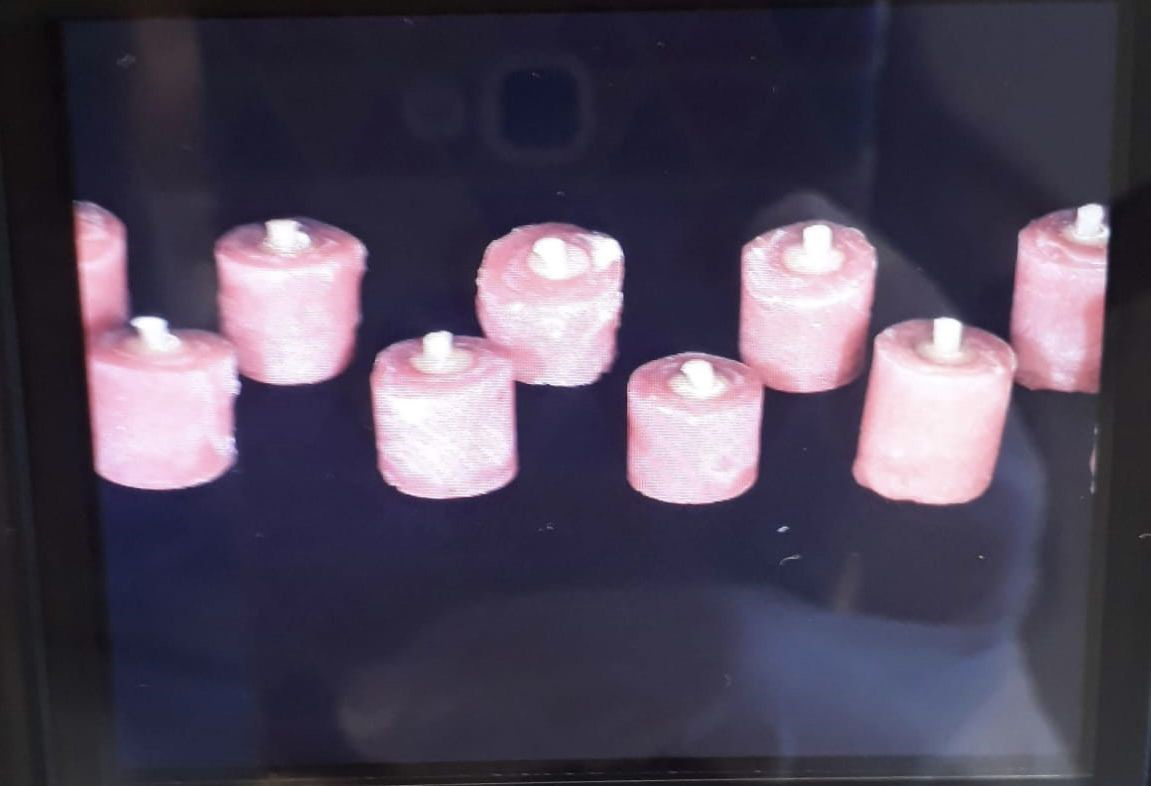
Specimens in used this study

Table 2 lists the surface treatment, silane, and adhesive resin application steps of the groups. After surface treatments and adhesive applications, transparent plastic molds with a height of 2 mm and a diameter of 2 mm were placed in the center of the samples. Then, the direct resin composite material was applied up to a thickness of 2 mm and cured with a curing device for 20 seconds (Figure 1). All specimens were kept in distilled water at 37°C for 24 hours after the transparent plastic mold was removed. Then, with a rest interval of 20 seconds in each bath, all groups were thermocycled for 5000 times in water baths between + 5°C and + 55°C.

**Table 2:** Surface conditioning procedures used in this study

**Groups**	**Application protocols**
1.Control [ER (etch and rinse mode of universal adhesive)]/ No surface pretreatment	Rinse surface with water and dry with a water-air spray. Apply phosphoric acid (FGM, Joinville, Brazil) for 30 seconds. Rinse and dry for 15 seconds. Apply I-BOND Universal (Heraeus-Kulzer, Hanau, Germany) by gently rubbing for 20 seconds and apply gentle air blast to evaporate solvent. Light cure for 10 seconds
2. SE (self- etch mode of universal adhesive)	Rinse surface with water and dry with a water-air spray Apply I-BOND Universal (Heraeus-Kulzer, Hanau, Germany) by gently rubbing for 20 seconds and apply gentle air blast to evaporate solvent. Light cure for 10 seconds.
3. Bur + ER (etch and rinse mode of universal adhesive)	Rough the surface with a fine grid diamond bur (20 strokes for each specimen) and change the bur in every 5 specimens. Rinse surface with water and dry with a water-air spray. Apply phosphoric acid (FGM, Joinville, Brazil) for 30 seconds. Rinse and dry for 15 seconds. Apply I-BOND Universal (Heraeus-Kulzer, Hanau, Germany) by gently rubbing for 20 seconds and apply gentle air blast to evaporate solvent. Light cure for 10 seconds.
4. Bur + SE (self- etch mode of universal adhesive)	Rough the surface with a fine grid diamond bur (20 strokes for each specimen) and change the bur in every 5 specimens. Rinse surface with water and dry with a water-air spray Apply I-BOND Universal (Heraeus-Kulzer, Hanau, Germany) by gently rubbing for 20 seconds and apply gentle air blast to evaporate solvent. Light cure for 10 seconds.
5. Bur + Silane + ER (roughen with bur + acid etching + silane + universal adhesive)	Rough the surface with a fine grid diamond bur (20 strokes for each specimen) and change the bur in every 5 specimens. Rinse surface with water and dry with a water-air spray Apply phosphoric acid (FGM, Joinville, Brazil) for 30 seconds. Rinse and dry for 15 seconds. Apply Dentobond silane (Itena, Paris, France) by gently rubbing for 30 seconds and apply gentle air blast to evaporate solvent. Apply I-BOND Universal (Heraeus-Kulzer, Hanau, Germany) by gently rubbing for 20 seconds and apply gentle air blast to evaporate solvent. Light cure for 10 seconds
6. Bur + Silane + SE (self- etch mode of universal adhesive)	Rough the surface with a fine grid diamond bur (20 strokes for each specimen) and change the bur in every 5 specimens. Rinse surface with water and dry with a water-air spray Apply Dentobond silane (Itena, Paris, France) by gently rubbing for 30 seconds and apply gentle air blast to evaporate solvent. Apply I-BOND Universal (Heraeus-Kulzer, Hanau, Germany) by gently rubbing for 20 seconds and apply gentle air blast to evaporate solvent. Light cure for 10 seconds.

###  Shear bond strength test

**Figure 2:**
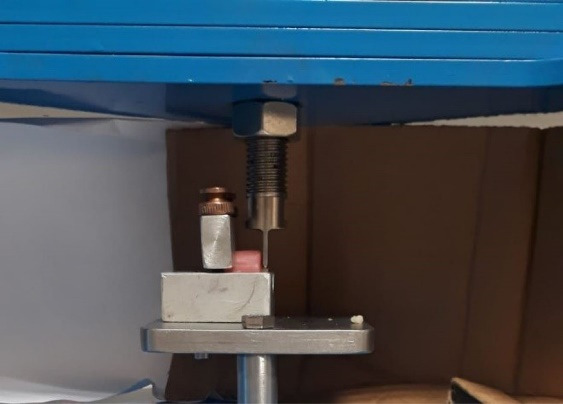
Testing procedure

 Shear bond strength tests were carried out using universal testing equipment with a crosshead speed of 1 mm/min. The force was determined in megapascals (MPa) by dividing the surface area of the applied resin composite by the maximum load causing bonding failure in Newton (N), (Figure 2).

 A stereo microscope (Euromax, Germany) was used to inspect surfaces at a magnification of 20 to determine the following types of failure modes: Type I: adhesive (between the substrate and repair composite), Type II: cohesive (within the substrate or within repair composite), and Type III: mixed (involving the interface and composite material).

###  Statistical analysis

 The data was analyzed using version 18 of the Statistical Package for Social Sciences (SPSS, Chicago, IL, USA). Two-way analysis of variance (ANOVA) was used to evaluate the data, and pairwise comparisons were done using the Bonferroni test at a significance level of 0.05.

## Results

 The result of ANOVA revealed that repair procedures and the type of resin composite significantly affect the bond strength values and that interaction is also significant (*P* = 0.00) (Table 3).

**Table 3:** Results of two-way analysis of variance (ANOVA)

	**SS**	**df**	**MS**	**F**	***P***
Corrected model	20207.208	17	1188.659	13.597	0.000
Intercept	189273.615	1	189273.615	2165.162	0.000
Surface treatment methods	20207.208	17	1188.659	13.597	0.000
Error	21679.604	248	87.418		
Total	230805.216	266			

**SS, **sum-of-squares; DF, degree of freedom; MS, mean squares.

Table 4 shows the standard deviations and mean shear bond strength values for each group.

**Table 4:** Means and standard deviations (Mean ± SD) of the repair bond strength values in all groups (Mpa)

**Composite**	**Surface Treatment Methods**					
	**ER**	**SE**	**Bur+ER**	**Bur+SE**	**Bur+Silane+ER**	**Bur+Silane+SE**
	**Mean±SD**	**Mean±SD**	**Mean±SD**	**Mean±SD**	**Mean±SD**	**Mean±SD**
Filtek Z250	29.70 ± 10.09^A^	20.73 ± 6.28^B^	29.53 ± 6.84^A^	34.08 ± 7.79 ^Ax^	13.21 ± 5.77^B^	44.80 ± 13.96^Cx^
Gradia Plus	25.87 ± 7.72^Ax^	22.69 ± 13.70^Bx^	28.72 ± 8.65^Ax^	24.76 ± 5.98^Bx^	13.44 ± 6.93^B^	26.99 ± 11.39^Cx**^
Ceramage	30.18 ± 10.65^Ax^	20.17 ± 7.95^B^	32.09 ± 8.57^Ax^	32.90 ± 9.45^Ax^	10.99 ± 5.33^B^	39.57 ± 13.53^Cx^

SD, standard deviation. *Note*: Statistically significant differences were demonstrated the difference letters in the rows. If the x mark is used in different letters, there is no difference in those groups shown on the rows. Statistically significant differences were demonstrated superscript by ** in columns (*P* < 0.05).

 The highest bond strength values were obtained for the Bur + Silane + SE application groups, while the lowest values were obtained for the Bur + Silane + ER application groups according to the tested repair procedures.

 In multiple comparisons, within the Filtek Z250 groups, Bur + Silane + ER application, with the lowest shear bond strength value, demonstrated a statistically significant difference from the other groups tested, except for the Bur + SE application group (*P* < 0.05). Statistically significant differences were observed between the Bur + Silane + ER group and ER (*P =*0.05), Bur + ER (*P =*0.02), and Bur + Silane + SE (*P =*0.014) application groups in Gradia Plus. The mean shear bond strength of the Bur + Silane + ER application group also exhibited the lowest value in Ceramage groups and showed significant differences from ER (etch&rinse group) (*P =*0.00), Bur + ER (*P =*0.00), Bur + SE (*P =*0.00), and Bur + Silane + SE (*P =*0.00) groups.

 The comparison of restorative materials revealed a statistically significant difference between Gradia Plus and the other resin composites tested in only the Bur + Silane + SE application groups. In other repair procedure groups, a significant difference between the restorative materials was not observed (*P* < 0.05).

**Figure 3:**
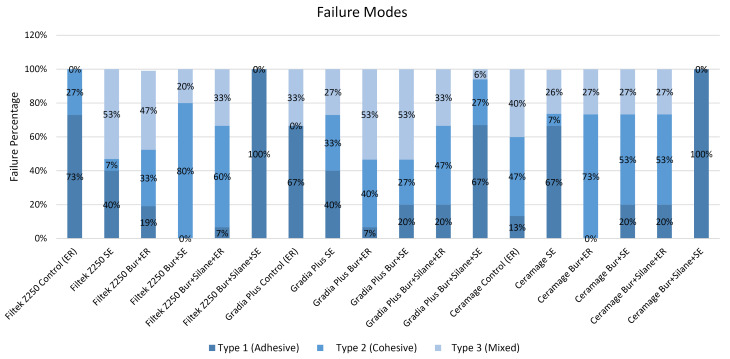
Distribution of failure modes

 The distribution of failure modes is illustrated in Figure 3. The adhesive failure mode was observed in the control [ER] groups of Filtek Z250 and Gradia Plus, in the Bur + Silane + ER groups of Filtek Z250, Ceramage, and Gradia Plus, and in the SE group of Ceramage, while the cohesive and mixed failure modes were predominantly observed in the other groups.

## Discussion

 In present study, with different repair processes, the repair capacities of two indirect resin composites (Gradia Plus and Ceramage, respectively) and a direct resin composite (Filtek Z250) were examined. The findings demonstrated that surface treatments and the kind of restorative material can alter the repair bond strength of direct and indirect resin composites. Therefore, H1 and H2 hypotheses are rejected.

 The indirect composite resins possibly exhibited a high degree of conversion and revealed a small number of free radicals due to the difference in polymerization methods.^3,13^ Although the high degree of conversion led to the improvement in the mechanical and physical properties of indirect composites, the bonding of a direct resin composite material on their surface in the repair procedure could be difficult.^3^ A successful repair procedure relies on the improved adhesion between the old restoration and the freshly added resin composite.^14^ Several techniques have been employed to enhance this adhesion, including micromechanical adherence and chemical bonding. Three techniques were used to secure the link between the aged composite and the new composite surface (micromechanical retention on the treated surface, chemical bonding with the exposed filler, and chemical bonding with the organic matrix).^2^

 Diamond bur, air abrasion, sandblasting, acid etching, and laser irradiation all induce micromechanical adhesion, which can improve the bond strength of the repaired composite.^2^ In this study, the etch&rinse and universal adhesive’s self-etch modes and diamond burs were utilized for surface pretreatment. Previous research^15-17^ has found that preparation of the resin composite’s surface with a diamond bur or sandblasting increases surface roughness and composite-to-composite repair bond strength. In addition, diamond bur application has been reported to render retentive properties at the micro and macro levels.^7^

 The preference of pretreatment procedures in the repair process was conducted according to the procedure that can be easily employed in daily practice. The unconverted carbon = carbon double bonds are related to chemical bonding on an organic matrix and an aged composite surface.^18^ Silane has proven to be very effective in chemical bonding with exposed filler particles to increase adhesion, and in creating a siloxane link between silicate-containing filler particles and the resin matrix.^3,19^ In addition, silane could make it easier for the bonding agent to penetrate irregular surfaces.^19^ In this study, although the mechanical surface treatment + universal adhesive’s self-etch mode in Filtek Z250 and Ceramage materials increases the shear bond strength, the mechanical surface treatment + universal adhesive’s etch&rinse mode did not affect the shear bond strength of all materials. In addition, silane application led to the increase in the shear bond strength in groups of self-etch mode of universal adhesive + mechanical surface treatment, while silane application led to the decrease in the shear bond strength in groups of the etch&rinse mode of the universal adhesive + mechanical surface treatment.

 Andrade et al^20^ evaluated the bond strength of mended on aged composite resin using a mixture of surface treatments and bonding agents. and found that the microshear bond strength of the aged Filtek Z250 composite is comparable to that of the mechanical surface treatment + etch&rinse group and that of the mechanical surface treatment + self-etch group. Kimyai et al^14^ have investigated the effect of three mechanical surface treatments (i.e., diamond bur, air abrasion, and Er, Cr: YSGG laser, respectively) on the repair bond strength of a Gradia indirect composite resin and reported that the shear bond strength of the Gradia indirect composite in the diamond bur + silane group is greater than that of the control + silane group. The microtensile bond strength of the Ceramage indirect composite can be improved by sandblasting with or without silane treatment, according to Visuttiwattanakorn et al.^2^ They also found that the bond strength of the sandblasting + ultrasonic clean group in Ceramage is lower than the bond strength of the sandblasting group alone. Burnett et al^21^ evaluated the tensile bond strength of a universal adhesive system on indirect composite surfaces treated with an Er:YAG laser, fluoridric acid, or air abrasion and reported that the tensile bond strength of the indirect composite is similar to that of the mechanical surface treatment + acid etching group between only the mechanical surface treatment group.

 In our study, silane application led to the increased shear bond strength in groups of the universal adhesive’s self-etch mode + mechanical surface treatment, while it led to the decreased shear bond strength in groups of etch&rinse mode of the universal adhesive + mechanical surface treatment. Moisture at the interface resulting from rinsing and drying after the acid etching of the repaired indirect resin composite might adversely affect the bonding of silane; hence, bond strength decreases. As a result, water absorption may reduce resin composite materials’ bonding ability and mechanical qualities.^21^ Another possibility is that the polar character of these adhesive systems’ phosphate groups contributes to the adherence of the inorganic load component of composite resins. When mechanical surface treatment and acid etching were performed on this adhesive system, chemical characteristics may be adversely affected.^20^ The discrepancy between our results and previous studies^2,13,14^ could be attributed to the different substrate, surface characteristics, and methodologies used in the study protocols. Moreover, differences in the adhesive system might demonstrate distinctive results as surface irregularities can be affected by the viscosity of the adhesive system and surface tension.^14^ Restorative materials are thought to have a desired feature of repairability. Mean bond strength values of greater than 18 MPa are required for a repaired composite to be clinically acceptable.^20^ In our study, bond strength values were acceptable, except for the Bur + Silane + ER groups of all the tested materials. Technique sensitivity may be seen in intraoral procedures with multiple phases.^22^ Therefore, the universal adhesive’s self-etch mode could simplify the bonding procedure during the repair of indirect and direct composite resins, shorten the procedure steps, and reduce technique sensitivity.^20^

 In this study, the bond strength of the Bur + Silane + SE group of Gradia Plus material was less than those of the Bur + Silane + SE groups of Ceramage and Filtek Z250. This result could be explained by the different filler composition, ratio between materials, and different abilities of surface treatments to expose silicate particles for chemical bonding with silane molecules.^23^ GC Gradia Plus (GC Inc., Kyoto, Japan) exhibits high strength, abrasion resistance, and superior polishability. It comprises a light-curing microfine ceramic/prepolymer filler with a urethane dimethacrylate (UDMA) matrix. Ceramage (SHOFU Inc., Kyoto, Japan) is as zirconium-silicate-integrated indirect restorative material. The progressively fine structure filler ratio in the organic polymer matrix ( > 73%) renders high flexural strength, elasticity, and ideal polishability to the structure,^24^ while Ceramage and Filtek Z250 comprise a zirconium silicate filler, and Gradia Plus does not comprise a zirconium silicate filler. The bonding agent used herein comprises a 10-methacryloyloxydecyl dihydrogen phosphate (MDP) monomer, and MDP monomers can chemically bond to a zirconia surface.^9^ In addition, it does not contain silane molecules, possibly explaining the lower bond strength of the Gradia Plus material compared with those of Ceramage and Filtek Z250.

 The lifetime of adhesive restorations is influenced by several factors. During aging, the resin matrix and/or resin filler interface absorb water molecules, causing the structure of the polymer matrix to degrade, transform to plastic, and hydrolyze.^9^ Furthermore, tension induced by repetitive heat expansion can compromise the adhesive interface’s strength.^9,25,26^ After the initial installation of restorations, intra-oral composite healing is performed for months or years.^27^ Therefore, to evaluate the success of repair procedures, the effect of aging on the resin matrix structure and repair interface should be considered.^9^ In this study, the aging process was applied to composite specimens before and after repair procedures were performed.^11,16^

 The shear bond strength test is the most common test used to evaluate bond strength, which is easily performed. Shear stresses are thought to cause major stresses in the failure of the restorative material under clinical conditions. Data obtained from the shear bond strength test were used for the comparison of material properties and the effects of surface treatment conditions that can enhance the fracture resistance.^28^ However, the macroshear bond strength test results revealed a nonuniform stress distribution, leading to more cohesive failures than those observed when conducting microshear and microtensile tests.^29^

 In this study, except for the control [ER] groups of Filtek Z250 and Gradia Plus, the Bur + Silane + ER groups of Filtek Z250, Ceramage, and Gradia Plus and the SE group of Ceramage, cohesive and mixed failure modes were predominantly observed, which was in agreement with previously reported results.^6,14^ Furthermore, contradictory findings about the failure modes have been reported in similar studies.^3,9,13^ These differences can be attributed to the materials and methodologies, and in fact, the repair procedures of resin composites can be affected by the surface treatment protocol.^3^

 Although repair bond strength values appear to be promising, the use of only one universal adhesive system could be one of the limitations of the study. Therefore, data obtained from this study may not be valid for all universal adhesive systems. Thus, further studies are required to implement standard repair protocols for direct and indirect resin composites with different components, test methods, and different surface pretreatment procedures. Careful case selection and appropriate use of surface treatment agents, followed by the use of a good-quality bonding system and restorative materials, can result in a repair that exhibits excellent retention and natural color blending.

## Conclusion

 Within the limitations of this in vitro study, the following conclusions can be made:

- The universal adhesive’s self-etch mode resin might be used to reduce adhesive application steps in the repair procedure of direct and indirect composites.
- An additional silane application step for universal adhesive’s self-etch mode might be recommended for resin composites containing zirconium silicate fillers.
- If the universal adhesive’s self-etch mode in Filtek Z250 and Ceramage materials is employed, roughening with bur can increase the shear bond strength.

## Acknowledgements

 The authors gratefully acknowledge Dr. Sevilay KARAHAN, Department of Biostatistics Faculty of Medicine, University of Hacettepe, for statistical support.

## Competing Interests

 The authors do not have any financial interest in the companies whose materials are included in this article.

## Ethical Approval

 Not applicable.

## Funding

 None.
